# [^68^Ga]Ga-FAPI-04 PET/CT in a patient with endometriosis: a potential game changer?

**DOI:** 10.1007/s00259-025-07252-9

**Published:** 2025-04-04

**Authors:** Caroline Burgard, Florian Rosar, Samer Ezziddin, Bashar H. Hamoud, Erich-Franz Solomayer, Matthias W. Laschke, Alin Constantin, Mark Bartholomä

**Affiliations:** 1 Department. of Nuclear Medicine, Saarland University – Medical Center, Kirrberger Str. 100, Geb. 50, 66421 Homburg, Germany; 2 Department of Gynecology and Obstetrics, Saarland University, Homburg, Germany; 3 Institute for Clinical and Experimental Surgery, Saarland University, Homburg, Germany


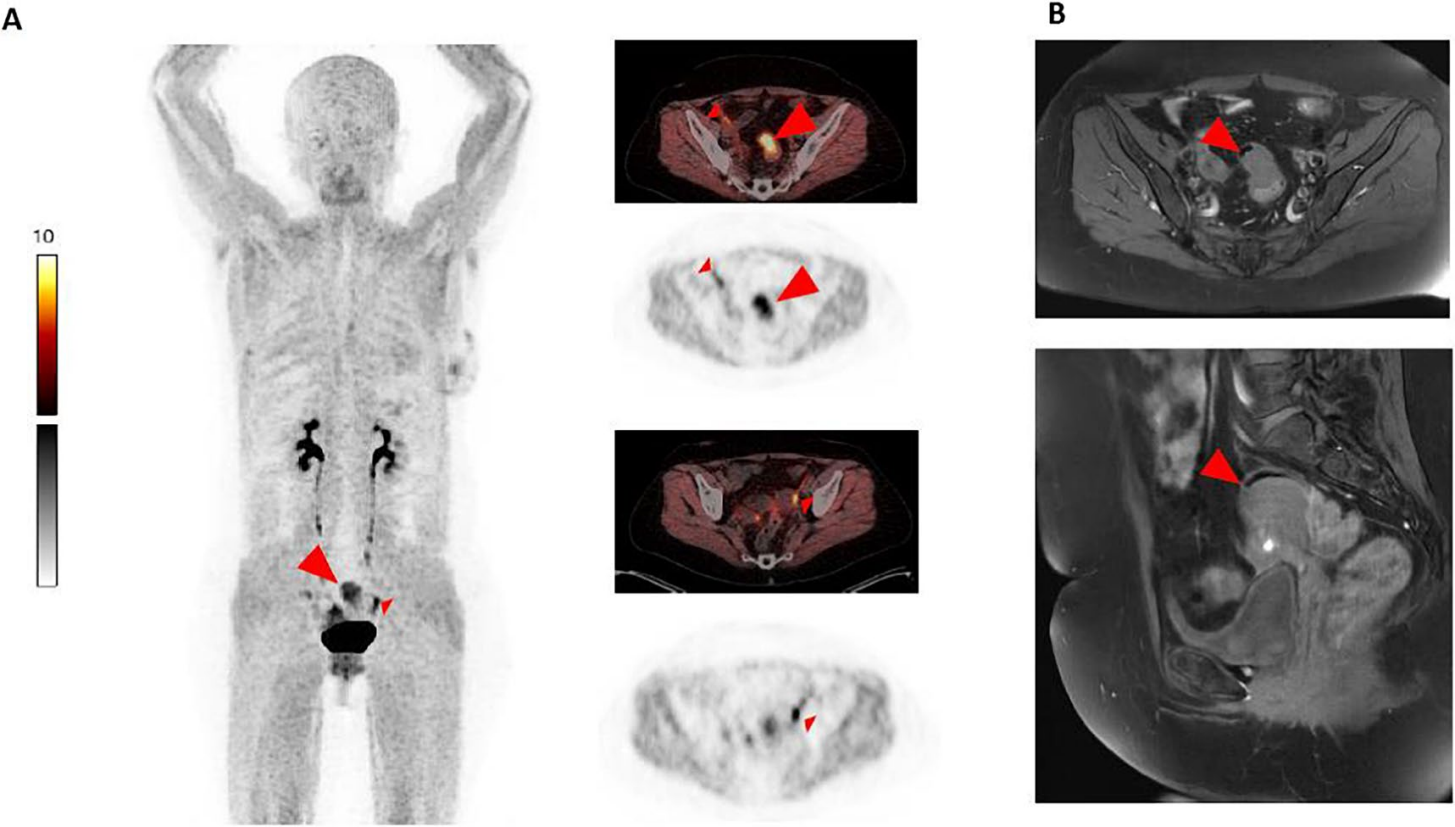


A 43-year-old woman with a known history of endometriosis presented with dysmenorrhea, dyschezia, and hematochezia. She had previously undergone a hysterectomy, during which infiltrative endometriosis in the bowel was identified. To guide further treatment decisions, a magnet resonance imaging (MRI) was performed, confirming bowel involvement and revealing a circumscribed lesion infiltrating the sigmoid colon. Due to persistent symptoms, the patient opted for surgery. For preoperative planning, a fibroblast activation protein inhibitor (FAPI) positron emission tomography/ computed tomography with [^68^Ga]Ga-FAPI-04 was conducted. PET images, acquired 10 min after administration 148 MBq of [^68^Ga]Ga-FAPI-04, showed intense tracer uptake (Fig. A; left column: maximum intensity projection; right column: axial slices of PET/CT fusion and PET; big arrow head: endometriosis in the sigmoid colon, SUVmax 9.8) at the same location as the MRI finding (Fig. B, axial and sagittal slices of contrast enhanced T1-weighted fat saturated sequence), thus confirming the MRI result. Additionally, the PET scan revealed intense uptake in the left ovary and the ligamentum teres uteri bilaterally (Fig. A, small arrowheads; SUVmax: 11.3, 7.3 and 5.4, respectively) suggesting the presence of additional endometriotic lesions undetected by MRI. The patient underwent a second surgical procedure, which histopathologically confirmed a deep infiltrating endometriosis extragenitalis.

Endometriosis, characterized by the ectopic growth of endometrial-like tissue, is a common gynecological disease affecting approximately 10% of reproductive-age women [1]. It can cause significant symptoms, including dysmenorrhea, dyspareunia, infertility, and gastrointestinal disturbances [2]. In addition to its physical effects, endometriosis can also affect mental health [3]. Thus, endometriosis significantly impact the quality of life of patients [4]. Diagnosis is primarily symptom-based, which can be challenging due to their variety and the heterogenic phenotype of endometriosis overlapping with other diseases. A definitive diagnosis is mostly confirmed through diagnostic laparoscopy. While transvaginal ultrasound is the first-line imaging modality, MRI is often used as a second-line tool for assessing disease extent and aiding in surgical planning [5].

This case highlights the potential role of ^68^Ga-FAPI PET/CT in detecting endometriotic lesions. Not only did the scan confirm the known lesion, but also identified additional sites of involvement undetected by MRI. Many studies have demonstrated the biodistribution of the ^68^Ga-labeled FAPI tracer, often showing intense uptake in the uterus [6–8]. There has been a previous case report showing only faint uptake in one endometriotic lesion [9]. To the best of our knowledge, this is the first confirmed case of endometriosis demonstrated on ^68^Ga-FAPI PET/CT with clear and intensive uptake. Therefore, ^68^Ga-FAPI PET/CT could represent a promising diagnostic modality for endometriosis, alongside many malignant diseases in which it has proven effective [10–13]. Our observation is supported by a recent preclinical study that found FAP expression in the immune microenvironment of endometriosis from various locations [14].

This case may provide a rationale for future studies systematically analyzing FAP expression in endometriosis across larger patient cohorts, investigating the value of ^68^Ga-FAPI PET/CT. A prospective study was recently initiated and is currently underway investigating this issue (NCT06792318).

## Data Availability

The data that support the findings of this study are available from the corresponding author upon reasonable request.
